# Different Approaches to Oral Lichen Planus Treatment: A Narrative Review

**DOI:** 10.3390/ijms27020914

**Published:** 2026-01-16

**Authors:** Irena Duś-Ilnicka, Andrzej Małysa, Marta Mazur, Anna Paradowska-Stolarz

**Affiliations:** 1 Division of General and Experimental Pathology, Department of Clinical and Experimental Pathology, Wroclaw Medical University, ul. T. Marcinkowskiego 1, 50-368 Wroclaw, Poland; 2 Department of Experimental Dentistry, Wroclaw Medical University, 50-425 Wroclaw, Poland; 3 Interdisciplinary Department of Wellbeing, Health and Environmental Sustainability-BeSSA Department, Sapienza University of Rome, 02100 Rieti, Italy; 4 Division of Dentofacial Anomalies, Department of Orthodontics and Dentofacial Orthopedics, Wroclaw Medical University, Krakowska 26, 50-425 Wroclaw, Poland

**Keywords:** oral lichen planus, pathology, oral, photochemotherapy, laser therapy, immunosuppressants

## Abstract

Oral lichen planus (OLP) is a chronic immune-mediated disorder affecting the mucous membranes of the oral cavity, characterized by inflammation caused by T-cell-mediated destruction of basal keratinocytes with the potential for malignant transformation. The exact etiology of the disease remains unclear, but as its symptoms may reduce patient quality of life, various treatment modalities have been proposed, generally based on managing symptoms and controlling disease progression. In this narrative review, we examine both conventional therapies (corticosteroids, immunosuppressants, retinoids) and emerging treatment options (photodynamic therapy, low-level laser therapy, and biologics) in terms of their efficacy and limitations. Although corticosteroid therapy remains a cornerstone of treatment, it is not effective in all cases, demonstrating the need to investigate alternative methods; hence, we also present possible future directions for OLP treatment in this study.

## 1. Introduction

Many systemic diseases, particularly autoimmune ones, manifest in the oral cavity and stomatognathic system [1,2,3,4]. Oral lichen planus (OLP) is an inflammatory condition of the oral mucosa affecting 0.5–2% of the global population [5,6], with a higher prevalence among perimenopausal women [6]. The condition is chronic [7] and has a low risk of progression to oral cavity cancer [8]; additionally, the differential diagnosis of OLP with leukoplakia is challenging. Interestingly, the use of fractal dimension and texture analyses may help differentiate the two diseases [9].

The pathogenesis of OLP is immune-mediated, with Cytotoxic T Lymphocyte Cells (CTLs), also known as Killer T (CD8+ T) cells, inducing apoptosis of basal keratinocytes [10,11]. Other inflammatory cytokines, such as TNF-1α (tumor necrosis factor-alpha), IL-17 (interleukin-17), and INF-γ (interferon-gamma), do not initiate the disease, but play roles in its progression [10]. Clinically, oral lichen planus presents in six primary forms, erosive, atrophic, reticular, papular, plaque-like, and bullous, with the reticular and erosive forms being the most common [12]. In most cases, reticular OLP is asymptomatic, while its erosive and atrophic forms may cause unpleasant symptoms such as pain and discomfort and present an increased risk of malignant transformations, potentially leading to oral squamous cell carcinoma (OSCC) [5,13].

Consequently, due to the multiple forms and presentations of the disease, treatment strategies differ. In most cases, they focus on modulating the immune response and relieving symptoms. In this review paper, we aim to highlight the most effective therapies for OLP, demonstrate conventional and emerging treatments, and outline potential future directions in the field.

## 2. Materials and Methods

For the purpose of this study, the Mendeley, PubMed and Scopus databases were searched first in January–March 2025, and then in November 2025. The search words and phrases were “Oral Lichen Planus” or “OLP” and “treatment”. Due to the variety of articles concerning different approaches to the treatment of the disease and the lack of repetitiveness, the authors concluded that it was impossible to prepare a systematic review on this topic. As a final result, the authors agreed that a narrative review would be prepared.

## 3. OLP Treatment Approaches

**Figure 1:**
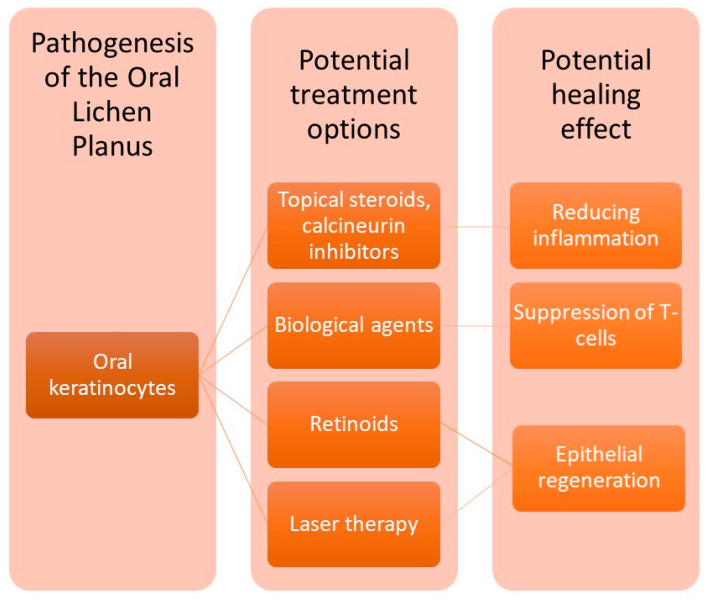
Available treatment possibilities based on OLP (oral lichen planus) pathogenesis.

OLP shows rare remission and often locally occurring lesions, highlighting the need for treatment protocols [14]. The conventional treatment methods are presented in Figure 1.

### 3.1. Corticosteroids

Corticosteroids are natural hormones that modulate autoimmune processes and are used to regulate inflammatory and autoimmunological processes either locally or systemically depending on symptom severity [15,16]. Topical corticosteroids (e.g., fluocinonide 0.05%, triamcinolone acetonide 0.1%, or clobetasol propionate 0.05%) are used for localized lesions, but most commercially available treatment options in this case must be used off-label; therefore, the dosage is hard to estimate [12]. In contrast, systemic treatments are reserved for refractory cases and may be accompanied by significant side effects (e.g., osteoporosis, adrenal suppression, hyperglycemia). In such cases, 30–60 mg of Prednisone/day may be effective [17]. Corticosteroids may also be used in combination with other immunosuppressive drugs, such as methotrexate and azathioprine, as well as biologic agents (e.g., efalizumab, alefacept) [18].

### 3.2. Retinoids

Synthetic vitamin A derivatives (retinoids) have recently been explored for the treatment of many diseases, including OLP, due to their role in epithelial differentiation and immune modulation. Local vitamin A supplementation has a notable effect on the oral epithelium, with topical retinoids (e.g., isotretinoin, tretinoin) being particularly effective in the treatment of oral cavity lesions. However, they may cause mucosal irritation [16]. Systemic retinoids (e.g., Acitretin) may be considered in refractory cases, but demonstrate significant side effects, including teratogenicity and hepatotoxicity [5]. It should also be noted that retinoids are the least effective medications for OLP treatment, so their use remains controversial [19].

### 3.3. Immunosuppressants and Calcineurin Inhibitors

In cases of corticosteroid resistance, other medicines, such as calcineurin inhibitors (including tacrolimus, pimecrolimus, and ciclosporin), may be used. This process, however, is not the first-line approach to treatment and requires active patient consent, as topical tacrolimus ointments are prescribed off-label [20]. This method of prescribing pharmaceuticals is often required during the treatment of oral pathologies, as few available drugs contain active substances that might have a curative effect on the affected oral mucosa, especially in OLP. The role of these substances is to suppress T-cell activation and cytokine release [21,22]; in these cases, topical application of 0.1% tacrolimus or 1% pimecrolimus may help resolve lesions. However, because of the limited accessibility of drugs containing these active substances, and as they are not specifically approved for this application (off-label use), their use should be carefully monitored. They may cause localized side effects during treatment, including burning mouth or concomitant opportunistic infections, and may increase the risk of malignancy when systemically absorbed; as such, they should be used as end-of-the-line treatment options [21,22,23,24].

## 4. Oral Cavity Sanitation

To reduce general symptoms, it is necessary for all patients with OLP to improve their personal oral hygiene and plaque control symptoms [14]. However, pathomorphological analysis of a biopsy is not always a first-line diagnostic test; for patients under the care of an oral pathologist, the first step should be to perform a thorough oral health evaluation and analyze all problems. All cavities should be filled, and the potential cause of the inflammation identified and documented. If any conservative/restorative fillings may need re-evaluation, the patient should be scheduled for a conservative dentistry visit to prevent harm to the oral mucosa. An observation period of 2 weeks to 1 month is required to evaluate whether the inflammation and disease have diminished [25].

## 5. Emerging and Adjunctive Therapies

Although the previous sections of this study have outlined the treatment options used to effectively manage the symptoms of OLP, there is no effective treatment option that offers lifelong relief [26]. In this section, we provide overviews of unconventional treatment methods that could be used in the treatment of OLP, most of which focus on relieving symptoms rather than addressing the cause of the disease.

### 5.1. Photodynamic Therapy (PDT)

Photodynamic therapy (PDT) uses a light-activated photosensitizer to selectively destroy inflammatory cells [26], potentially leading to the healing or proliferation of healthy tissue. PDT generates reactive oxygen species (ROS) to induce apoptosis of the inflammatory cells. Although several studies have reported that PDT can reduce pain and lesion size, the truth remains disputable [27,28,29]. Although the list of side effects is minimal, adverse reactions such as pain, swelling, and redness have been reported, eventually decreasing with subsequent treatment sessions [30]. Further limitations of this method include the small number of individuals examined and the need for specialized equipment, making it both expensive and limited in availability [30].

### 5.2. Low-Level Laser Therapy (LLLT)

Low-level laser therapy (LLLT) is a novel treatment method based on CO_2_ that utilizes diode laser ablation. Laser therapy induces photobiomodulation, which reduces inflammation, acts as an anesthetic, and improves tissue regeneration and healing [31]. This method has the advantages of non-invasiveness and minimal side effects [31], as the reaction enhances mitochondrial activity and further reduces inflammation [29], a mechanism frequently used in bone healing [32]. Even though there is clinical evidence of symptomatic improvement in OLP patients treated with LLLT [31], the groups who received the treatment were relatively small, with a short postoperative follow-up. There is no established protocol, and the true efficacy of the method is unclear due to the use of varying laser wavelengths (630–808 nm) [31]; the most commonly used forms are Helium–Neon (632 nm) and, more recently, diode lasers (600–1100 nm). It is also possible to use ultraviolet lasers, with waves below 350 nm in length. Treatment outcomes depend on wavelength, intensity, power, duration, and number of sessions. According to the latest suggestions from the World Federation of Laser Therapy (WFLT), the optimal wavelengths for mucosal treatment are 630–670 nm when applied intraorally and 780–850 nm when applied extraorally. Furthermore, the process also remains expensive due to the need for specialist devices. Despite these limitations, photostimulation remains a promising method for treating OLP [33,34,35].

### 5.3. Biologic Agents

A new and frequently discussed method of treating autoimmune conditions is the use of biologic agents. As such, targeted immunotherapy has emerged as a treatment for OLP. TNF-α inhibitors, such as adalimumab and infliximab, may reduce inflammation through neutralization [17]. Another option is the use of IL-17 inhibitors, such as secukinumab, which target the key cytokine in OLP etiology [36]. These treatments may be effective because they aim to reduce the cause of the disease, not the symptoms. However, problems arise when they cannot act appropriately or fully. Preliminary studies suggest that biologic agents might be effective, but the small number of participants in these studies indicates the need for further research.

### 5.4. Herbal and Natural Therapies

Due to the strong trend toward the use of natural substances and “green dentistry” [33,36], there is a great need to develop related treatment methods. Therefore, herbal agents are sometimes used in the treatment of OLP for their anti-inflammatory and immunomodulatory properties, including aloe vera, which has been shown to reduce lesion size and decrease pain [37]; curcumin, which exhibits antioxidant and anti-inflammatory properties [38]; and green tea extract, which possesses an immunosuppressive effect and is rich in polyphenols [39]. These treatments seem promising, but further clinical trials with larger patient numbers are necessary, as most research has been conducted with small sample sizes.

The use of flaxseed (*Linum usitatissimum* L.) has also been studied, both as oil extracts applied directly to the oral mucosa and in the form of ointments that patients can produce at home, as previously confirmed using cell cultures of oral keratinocytes [40]. As oral ointments and artificial saliva products may represent an economic burden for patients, flaxseed may be used to alleviate burning and dryness of the oral mucosa instead. It has been previously confirmed that the active substances in flaxseed may act on the oral mucosa and have a possible relationship with nuclear receptors for estrogens, making them particularly suitable for phytotherapy to support perimenopausal women with OLP [40,41,42].

As previously mentioned, improving oral hygiene habits also reduces inflammation [14]. In this case, using less-irritating, more natural substances in daily oral care might be the key to managing small to moderate changes in the oral cavity [43].

## 6. Biomarkers Related to OLP Diagnostics

In the field of laboratory medicine, a variety of diagnostic tools are available, including biochemical, immunological, molecular biology, and histochemical biomarkers. By analyzing blood, saliva [44], and crevicular fluid, they can be used to determine the progression of the disorder [45], remission [46], genetic status, other diseases related to OLP, and infections [47]. OLP can also be predicted by measuring peroxidation biomarkers, antioxidants, levels of hormones such as cortisol, and indicators of the immune response, such as immunoglobulins [48].

Diabetes mellitus has also been associated with OLP [48,49], and is classified as either Type 1 (T1DM) or Type 2 (T2DM). T1DM results from an autoimmune-induced insulin deficiency, while T2DM results from insulin resistance [49]. For this reason, accurate daily blood glucose monitoring and glycated hemoglobin (HbA1c) every 2–3 months are essential for managing disease status [50].

Some studies have found associations between OLP and infectious diseases such as HBV and HCV [42], although other reports have also noted no correlations [51,52]. For the relevant diagnostics of these parameters, patients with Hepatitis B can be provided with HBsAg/Anti-HBs/Anti-HBc to determine the acute, chronic, or immune status of the disease, and Anti-HCV/HCV RNA can be used to confirm active Hepatitis C infection. Possible biomarkers are presented in the Table 1.

Another widely discussed parameter, often analyzed in the saliva, is cortisol [53], which has been associated with stress levels in diverse OLP patient groups and may serve as a potential biomarker of OLP lesion development [48]. Another immunological salivary marker is metalloproteinase MMP-8, which has been associated with disease status [54]. Although widely researched, interleukin 1 beta (Il-1β) status and prostaglandin (PGE) analysis in both saliva and serum have not been confirmed as relevant to diagnostics.

To date, the only confirmed diagnostic parameter for OLP is histopathological analysis to identify hyperkeratosis on hematoxylin–eosin-stained biopsy slides, although subepithelial, band-like lymphocytic infiltration remains the most characteristic finding. Other findings include thickening of the stratum granulosum, acanthosis of the spinous layer, and vacuolar degeneration of basal layer cells and the basement membrane. When extending to the epithelium, this may resemble a “saw-tooth” pattern, with an increased number of intraepithelial T-cells and colloid bodies in the lower epithelial layers and lamina propria. Other diagnostic methods include the iodine contact test, which has been used for semi-confirmatory diagnosis in pathologically altered oral mucosa; Lugol’s staining, an adjunctive tool that cannot replace histopathological examination by biopsy or assessment of malignant potential [55,56]; and toluidine blue, which shows similar efficacy to Lugol but binds to nucleic acids and accumulates in the areas of increased cellular proliferation. However, the latter may be more efficacious for differentiating malignant transformations [57]. This approach is motivated by the ongoing search for a noninvasive diagnostic test, especially in oral medicine, a neglected and financially inaccessible area of health care for many patients [58]. Based on the immunological approach and changes in the oral mucosa, as well as on direct immunofluorescence and immune function in patients with OLP, these topics were thoroughly discussed and evaluated. Direct immunofluorescence (DIF) has previously been used to study immune function in OLP [58,59] but remains an adjunctive technique not used in daily diagnostics.

## 7. Markers of Potential Malignant Transformation of OLP

Malignant transformation is observed in 1% of OLP cases, though there is no consensus in the field of oral pathology regarding the biomarkers that can be used to identify this transition [60]. OLP is also often a lifelong disorder, requiring recurrent checkups. As such, there is a need to develop biomarkers that can be used between checkups to reduce the need for dental visits.

First, alterations in apoptosis-related markers may represent the onset of malignancy [61]. Changes in the intrinsic pathway might be induced by hypoxia or free radicals, leading to the increased permeability of mitochondrial pores. Proteins that could potentially be included in this diagnostic approach include B-cell lymphoma protein 2 (BCL-2), BCL-2 antagonist killer 1 (BAK), BCL-2 antagonist of cell death (BAD), and BH3 domain death agonist (BID), as described by Tampa et al. [61]. The extrinsic pathway may also be triggered during apoptosis of the oral mucosa, primarily by tumor necrosis factor (TNF) [17].

Survivin, less often used in the field of oral pathology, is an Inhibitor of Apoptosis protein (IAP) and is expressed in Adult Stem Cells (ASCs) [62]. It is reported to be explicitly involved in carcinogenesis and is overexpressed in most cancers due to its contribution to clonal expansion [62]. Therefore, it indicates a risk of malignant transformation, as its expression increases with disease severity, and it may be associated with dysplastic OLP. For this reason, it should be thoroughly evaluated as a potential biomarker for identifying high-risk lesions [60,63].

The nuclear antigen Ki-67 is another marker that can be used to assess the risk of progression in oral lichen planus [64]. It is a well-established diagnostic marker of cell proliferation, has been widely studied in OSCC and precancerous oral lesions [64], and effectively diagnosed cancer-associated fibroblasts in metastatic oral tongue squamous cell carcinoma (SCC) [65].

Another biomarker with potential to highlight metastasis, p16 (p16INK4a), is used in the diagnostic process via immunohistochemistry and is recommended by both the American Society of Clinical Oncology (ASCO) and the American Joint Committee on Cancer (AJCC). This protein can be used to determine Human Papillomavirus (HPV) status in oropharyngeal squamous cell carcinoma (OPSCC) [66] and has been found to be more prevalent in OLP biopsies, in addition to diagnosing HSV, which is most prevalent in the buccal sites and gingiva of OLP-affected patients. Although this parameter is not directly related to ongoing HPV infection, it is essential to follow up on the pathological process potentially induced by the virus [67,68]. Possible immunohistochemical diagnostic approaches also include concomitant assessment of p53, which is associated with the potential for malignant transformation to oral cancer [60,69,70].

MicroRNAs are a group of biomarkers that draw research interest but are not commonly used in diagnosis, as they are not able to assess changes in the oral mucosa. These single-stranded RNAs are targeted through their 3′-untranslated region (3′-UTR), and are linked to inflammatory and immune diseases through the dysregulation of target mRNA expression [71]. In the current literature, several microRNAs have been assessed, including microRNA-146a, microRNA-155 [71], microRNA-4484, microRNA-21, and microRNA-142-3p, which may be upregulated in the saliva of patients with OLP, and microRNA-125a, microRNA-137, microRNA-320a, and microRNA-27b, which might be downregulated [72]. The aforementioned downregulated microRNAs play specific roles in the cell cycle as pathogenesis progresses. MicroRNA-125a has been evaluated as a tumor suppressor and, as such, is underexpressed in patients with OSCC [72]. The evaluation of microRNA-137, which is also downregulated in the saliva and tissues of patients with OLP, showed the potential to transform OLP into OSCC [72]. MicroRNA-320a has been identified as a potential therapeutic target for OLP [73]. It has been claimed that salivary levels of microRNA-320a, along with the highly sensitive C-reactive protein, could be used as a noninvasive predictive tool for cases of OLP in which dysplasia is observed [73]. Conversely, microRNAs upregulated in the saliva of patients with OLP may serve other diagnostic functions. The first example, related to an exon of the B-cell integration cluster gene, is microRNA-155, which is associated with immunological regulation and the autoimmune process; a potential association between upregulation of salivary microRNA-155 and OLP status was described by Tao et al. [71]. Exosomal microRNA-4484, also upregulated in OLP [72], was associated with potential pathological stimuli, such as cancer, and with inflammatory processes related to bacterial infection [72,74]. MicroRNA-21 was found to be upregulated in patients with OSCC compared with those with OLP [74], but its specific mechanisms of action have not yet been thoroughly investigated in this regard.

## 8. Future Directions and Challenges

Despite the vast number of potential diagnostic tools for OLP, given the risk of progression to malignancy, it should still be differentiated from other similar oral mucosa changes. True oral lichen planus is a condition with a strong autoimmunologic background but no clear cause, while oral lichenoid lesions (OLLs) might mimic the same clinical outcome, but possess a clear etiology and are based on external factors such as medication, dental materials (metals, resins), food, and other potential irritants. Although both conditions share similar clinical and microscopic features, OLLs are potentially easier to reverse upon removing the triggering factor due to their external etiology. However, the fact that a risk of malignant transformation still exists should not be ignored [75,76].

Despite significant advances in OLP treatment, corticosteroids remain the primary treatment for this condition. The importance of treating oral lichen planus is high, as it is potentially malignant [39], with a risk of progression to OSCC (oral squamous cell carcinoma) at the estimated rate of 1% [60]. The other authors [13] report that it even ranges up to 2%. Treatment for this condition is complex due to several challenges that must be addressed: a lack of curative therapy, the need for personalized medicine, and the need to develop novel targeted therapies. Current treatment methods do not actually cure the underlying disease but focus on relieving the patient’s symptoms. The problem lies in the lack of actual knowledge regarding OLP and the targeting of treatment, as in any other autoimmune disease. Genetic and immunologic factors also influence treatment response; therefore, there is no “standard” treatment, highlighting the need for individualized therapy. In the future, the development of novel targeted therapies (e.g., cytokine inhibitors and immune checkpoint modulators) may improve treatment effectiveness. Furthermore, a better understanding of the fundamentals underlying OLP may inform future treatments. Additional therapies have not yet been explored, but the use of natural treatments, such as aloe vera and herbal extracts, and the role of selenium and vitamin D dosage are still unclear [77,78,79].

Some studies have found that amalgam dental fillings should be removed to prevent changes in the oral cavity, including OLP. Still, recent studies do not show a relationship between these changes and the presence of such fillings [38], making the overall picture uncertain.

In addition to the lack of knowledge regarding its origins, a significant limitation in introducing standardized treatment methods for OLP is, in most cases, a lack of clear diagnostic criteria to identify the condition. A potentially interesting, non-invasive method to address this problem is fractal dimension analysis [9], but this might cause difficulties for clinicians and inexperienced doctors. Finally, it should be noted that OLP may accompany other diseases, making it more difficult to detect.

## 9. Conclusions

OLP causes many difficulties in treatment, and therefore, it is unclear whether a definitive cure exists. In most cases, treatment is local and symptomatic and may not eliminate the underlying systemic disease. Molecular diagnostic approaches are still underrepresented, as the molecular processes underlying cell structure pathogenesis are unclear. Corticosteroid use remains the primary and most effective treatment, but in future, emerging therapies such as PDT, LLLT, and biologics could be utilized as cures. Therefore, further research and a personalized treatment approach are needed to address the symptoms of OLP and its underlying cause. Immune-modulating therapies should remain a mainstay of treatment. Additional studies are also necessary to determine which treatment methods can be used interchangeably, as most alternative methods studied were trialed on a small number of participants.

## Figures and Tables

**Table 1:** Biomarkers related to OLP status which support diagnosis.

Biomarker	Diagnostic Parameter	Biomaterial	Supportive Diagnostic for OLP	OLP Diagnostic
**Glycated hemoglobin (HbA1c)**	Glucose	Blood	Yes	No
**Fasting (pre-meal) glucose levels**	Glucose	Blood	Yes	No
**HBsAg/Anti-HBs/Anti-HBc**	Hepatitis B	Serum	Yes	No
**Anti-HCV/HCV RNA**	Hepatitis C	Serum	Yes	No
**ELISA and immunological diagnostics for cortisol**	Cortisol	Saliva	Yes	No
**ELISA and immunological diagnostics for MMP-8**	MMP-8	Saliva	Yes	No
**Histopathologic diagnostics**	Oral keratinocytes	Biopsy	No	Yes

## Data Availability

No new data were created or analyzed in this study. Data sharing is not applicable to this article.
